# BMP7 and EREG Contribute to the Inductive Potential of Dental Mesenchyme

**DOI:** 10.1038/srep09903

**Published:** 2015-05-08

**Authors:** Bo Gao, Xin Zhou, Xuedong Zhou, Caixia Pi, Ruoshi Xu, Mian Wan, Jing Yang, Yue Zhou, Chengcheng Liu, Jianxun Sun, Yan Zhang, Liwei Zheng

**Affiliations:** 1 State Key Laboratory of Oral Diseases, West China Hospital of Stomatology, Sichuan University, Chengdu, Sichuan 610041, China; 2 West China School of Stomatology, Sichuan University, Chengdu, Sichuan 610041, China; 3 Department of Orofacial Sciences, University of California, San Francisco, CA 94143, USA; 4 Center for Craniofacial Regeneration (CCR), College of Dental Medicine, Columbia University Medical Center, New York, NY 10032, USA

## Abstract

Odontogenesis is accomplished by reciprocal signaling between the epithelial and mesenchymal compartments. It is generally accepted that the inductive mesenchyme is capable of inducing the odontogenic commitment of both dental and non-dental epithelial cells. However, the duration of this signal in the developing dental mesenchyme and whether adult dental pulp tissue maintains its inductive capability remain unclear. This study investigated the contribution of growth factors to regulating the inductive potential of the dental mesenchyme. Human oral epithelial cells (OEs) were co-cultured with either human dental mesenchymal/papilla cells (FDPCs) or human dental pulp cells (ADPCs) under 2-dimensional or 3-dimensional conditions. Odontogenic-associated genes and proteins were detected by qPCR and immunofluorescence, respectively, and significant differences were observed between the two co-culture systems. The BMP7 and EREG expression levels in FDPCs were significantly higher than in ADPCs, as indicated by human growth factor PCR arrays and immunofluorescence analyses. OEs co-cultured with ADPCs supplemented with BMP7 and EREG expressed ameloblastic differentiation genes. Our study suggests that BMP7 and EREG expression in late bell-stage human dental papilla contributes to the inductive potential of dental mesenchyme. Furthermore, adult dental pulp cells supplemented with these two growth factors re-established the inductive potential of postnatal dental pulp tissue.

Odontogenesis is accomplished via reciprocal signaling between the epithelium and the mesenchyme^1^,^2^. In the initiation stage, the odontogenic potential resides in the epithelial compartment and induces the presumptive dental mesenchyme to express odontogenesis-related genes^3^,^4^,^5^. After E12.5 in mice, the odontogenic potential shifts into the mesenchymal compartment and then reciprocally cross-talks with the epithelium. This inductive mesenchymal compartment is capable of determining the odontogenic fate of both dental and non-dental epithelium^3^,^6^,^7^,^8^,^9^,^10^,^11^. The latter will then commit to an odontogenic fate and eventually form enamel-forming ameloblasts.

The ameloblastic differentiation of dental epithelial cells is critical for enamel formation. The proliferating dental epithelial cells become the inner enamel epithelium and subsequently differentiate into preameloblasts, presecretory ameloblasts and then secretory ameloblasts that synthesize and secrete enamel matrix proteins. The enamel matrix proteins self-assemble to form a matrix, which mineralizes as the ameloblasts continue to differentiate^12^. During differentiation, epithelial cells are adjacent to different extracellular microenvironments and are thus exposed to different signals. In the human tooth, the basement membrane that separates the early developing dental epithelium and mesenchyme is lost shortly before dentin deposition is initiated and before enamel matrix secretion. Basement membrane proteins and signals from underlying mesenchymal cells coordinate the initiation of preameloblast differentiation^13^.

In a recent study, conserved odontogenic potential was studied in embryonic dental tissues^14^. This previous study demonstrated that human dental mesenchyme from the bell stage, but not from the cap stage, could induce mouse embryonic second-arch epithelium, as well as human keratinocyte stem cells, to become enamel-secreting ameloblasts. The authors also showed that neither postnatal human dental pulp stem cells (DPSCs) nor stem cells from human exfoliated deciduous teeth (SHED) possess odontogenic potential or are odontogenic competent. However, the underlying mechanism of the difference between embryonic dental tissue and adult dental tissue has yet to be elucidated.

Our previous study showed that human embryonic stem cell-derived epithelial cells (ES-ECs) are similar to ameloblast-lineage cells^15^ and that the developing human dental mesenchyme from the bell stage possesses the required signal to induce these ES-ECs to differentiate into dental epithelial cells^16^. This and other previous investigations have broadened the possibilities of cell-based enamel regeneration. Because of ethical considerations, it is impractical to use heterologous tissue from mouse or developing human dental papilla tissue for the tissue engineering of human teeth. Thus, a postnatal cell source is necessary for applications of enamel/tooth regeneration in humans. To date, little is understood about the potential for postnatal dental mesenchymal cells to drive ameloblastic differentiation of epithelial cells. However, numerous studies have proven that re-association of dissociated epithelial and mesenchymal compartments can form anatomically correct dental tissue^9^,^10^,^11^,^17^, providing sound evidence that tooth regeneration is possible through recombination of the epithelial-mesenchymal compartment.

Growth factors, including transforming growth factor β (TGFβ), fibroblast growth factors (FGFs), Notch, Sonic hedgehog (SHH), and Wnt family proteins, have been shown to mediate interactions between the dental epithelium and the mesenchyme^18^,^19^,^20^,^21^ and to play critical roles in organ development^22^,^23^,^24^,^25^. Although the relevant molecular mechanisms are not fully understood, these growth factors have been suggested to play a crucial role in odontogenesis,^18^,^26^,^27^,^28^. Based on the existing knowledge that re-associated dental epithelial cells can undergo morphogenesis under the inductive signal of the mesenchyme^29^, even after the complete loss of spatial information^30^, we hypothesized that cultured late bell-stage dental mesenchymal cells still possess inductive potential for non-dental epithelial cells after the complete loss of spatial information and that certain growth factors are responsible for this process.

Here, we examined differentially expressed growth factors in human dental papilla cells and human dental pulp cells and identified BMP7 and EREG as potential contributors to the odontogenic inductive potential of developing dental papilla. The addition of these growth factors to adult dental pulp cells allows this more accessible cell type to stimulate the differentiation of non-dental oral epithelial cells into ameloblast-lineage cells.

## Materials and Methods

### Ethics statement

All human tissues were collected from the West China Women and Children’s Hospital in accordance with the guidelines issued by Sichuan University. For the use of tissue samples, written informed consent was obtained from all human subjects who participated in the investigation. The study and consent procedures were approved by the ethical committees of the West China School of Stomatology, Sichuan University and the State Key Laboratory of Oral Diseases.

### *In vitro* two-dimensional (2-D) co-culture of human OEs and human dental papilla/pulp cells

Human dental papilla/pulp cells and oral epithelial cells were cultured as previously described^31^, and human fetal dental papilla cells (FDPCs) and adult dental pulp cells (ADPCs) were harvested. To limit their mitotic ability, the cells were placed on ice and transferred into an irradiator^32^,^33^. After irradiation with 8000 rads, the FDPCs and ADPCs were re-plated and grown to 40–50% confluency before co-culture with human oral epithelial cells (OEs). The next day, the OEs (2.5 × 10^4^ cells/cm^2^) were seeded onto either FDPCs (2.5 × 10^4^ cells/cm^2^) or ADPCs (2.5 × 10^4^ cells/cm^2^), and the cells were co-cultured for 1 week before harvesting. After enzymatic digestion for 1 min with 0.5% trypsin/EDTA at 37 °C, the enzymatic activity was quenched by culture medium containing serum, and then the mesenchymal cells were thoroughly washed 3 times with cold PBS to lift them off the plate. The epithelial clones were located microscopically and mechanically loosened using a 1000-μl pipette tip. The culture dish was then washed with culture medium, and the epithelial cells were collected and centrifuged to harvest the cell pellets. Total mRNA was purified using an RNeasy Micro kit (Qiagen, Valencia, CA, USA), and cDNA was synthesized using the SuperScript^TM^ III First-Strand Synthesis System (Invitrogen, Carlsbad, CA, USA). RT-PCR was performed to validate the presence of Vimentin and K14 transcripts in the collected cells. Then, qPCR was used to quantify the gene expression of amelogenin (AMLX, Applied Biosystems product number Hs00365791_m1), ameloblastin (AMBN, Applied Biosystems product number Hs00212970_m1), amelotin (AMTN, Applied Biosystems product number Hs00418384_m1), and cytokeratin 14 (KRT14, Applied Biosystems product number Hs00265033_m1). The qPCR conditions were as follows: 50 °C for 2 min and 95 °C for 10 min, followed by 40 cycles of 95 °C for 15 s and 60 °C for 1 min. The endogenous control was 18S rRNA, and mRNA expression levels were compared using the ΔΔCt method^34^. The data were compared by one-way ANOVA, followed by post hoc Tukey’s test.

### *In vitro* three-dimensional (3-D) co-culture of human OEs and human dental papilla/pulp cells

Matrigel^TM^ (BD Biosciences, San Jose, CA, USA) was thawed on ice at 4 °C overnight. The next day, human OEs (1 × 10^5^ cells) and human dental mesenchymal cells (1 × 10^5^ cells) were harvested and re-suspended in Matrigel^TM^. The Matrigel^TM^/dental mesenchymal cell complex was seeded onto a Transwell insert (Fisher Scientific, Pittsburgh, PA, USA), and the Matrigel^TM^/OE complex was seeded on top. The co-culture complex was maintained in a 37 °C incubator for 30 min to allow the Matrigel^TM^ to set. KGM-2 medium was added to the upper chamber of the Transwell apparatus, and DMEM was added to the bottom chamber. The co-culture complex was cultured for 1 week before 1 mM CaCl_2_ was added to the KGM-2 medium. Subsequently, 10 nM dexamethasone, 50 μg/ml ascorbic acid and 10 mM b-glycerophosphate were added to the DMEM^35^. Then, the complex was co-cultured for an additional 8 weeks.

### Histological analysis of the co-culture complex

After 3-D co-culturing, the Matrigel^TM^/cell complex was embedded in OCT compound (Sakura Finetek, Torrance, CA, USA) and then cryosectioned at a thickness of 10 μm and stored at −80 °C.

For immunofluorescence, the cryosections were retrieved from −80 °C and incubated in 3% goat serum, 0.1% BSA and 0.1% Triton (blocking solution) for 1 hr at room temperature. The sections were incubated with primary rabbit anti-amelogenin antibody (1:1000, Santa Cruz Biotechnology, Inc., Santa Cruz, CA, USA) overnight at 4 °C. After being washed thoroughly, the sections were incubated with a second primary antibody, mouse anti-cytokeratin 14 (1:200, Santa Cruz Biotechnology, Inc.), for 1 hr at room temperature. After being washed thoroughly, the sections were incubated with both FITC-conjugated anti-rabbit (1:160, Santa Cruz Biotechnology, Inc.) and TRITC-conjugated anti-mouse IgG (1:200) (Sigma-Aldrich, St. Louis, MO, USA) secondary antibodies for 1 hr. Nuclei were counterstained with 0.5 μg/mL Hoechst 33342 (Invitrogen Corporation) in the dark for 5 min. After mounting, the tissue sections were imaged using a Nikon Eclipse 300 fluorescence microscope (Compix Inc., Sewickley, PA, USA).

### Differential growth factor expression profiles of FDPCs and ADPCs by qPCR and qPCR arrays

TaqMan array plates (TaqMan® Array Human Growth Factors 96-well Plate) were purchased from Applied Biosystems. RNA was isolated and purified from cultured FDPCs and ADPCs using an RNeasy Mini kit (Qiagen, Valencia, CA, USA), and cDNA template was synthesized using the SuperScript^TM^ III First-Strand Synthesis System (Invitrogen, Carlsbad, CA, USA). Aliquots of cDNA (55 ng/reaction) were then mixed with an equal volume of TaqMan Gene Expression Master Mix (Applied Biosystems), and real-time PCR was performed using a 7900 FAST Real-Time PCR System (Applied Biosystems). The data were analyzed using DataAssist™ software (Invitrogen), and mRNA expression was compared by ΔΔCt^34^. The data were compared by one-way ANOVA.

The PCR array data were validated by real-time qPCR. Briefly, primary FDPCs and ADPCs were harvested, RNA was isolated and purified, and then cDNA was synthesized. The expression levels of BMP7 and EREG were examined by quantitative real-time PCR using an ABI 7900 system (Applied Biosystems, Foster City, CA, USA). The results were then normalized to the expression of GAPDH, which was used as an endogenous control, and mRNA expression levels were compared by ΔΔCt^34^. The data were compared by one-way ANOVA.

Human tooth buds were obtained from 18- to 22-week-old fetal cadavers within 3 hr after legal abortion. Adult dental pulp tissues were immediately collected from extracted third molars under a laminar flow hood. Mandibular tissue containing tooth buds from fetal cadavers and dental pulp tissue from adult third molars were fixed in 4% PFA at 4 °C overnight and then preserved in 20% sucrose solution for 6 hr at 4 °C before being embedded in OCT compound (Sakura Finetek, Torrance, CA, USA). The samples were then cryosectioned at a 6-μm thickness. For immunofluorescence, the cryosections were incubated in 3% goat serum, 0.1% BSA, and 0.1% Triton (blocking solution) for 1 hr at RT. The sections were incubated with primary rabbit anti-BMP7 (1:100) or anti-EREG (1:200) antibody overnight at 4 °C. After being washed thoroughly, the sections were incubated with FITC-conjugated anti-rabbit secondary antibody (1:160) (Sigma-Aldrich, St. Louis, MO, USA) for 1 hr. Nuclei were counterstained with 0.5 mg/mL Hoechst 33342 (Invitrogen Corporation) in the dark for 5 min, and then the slides were mounted. Morphometric analysis was performed using an image auto-analysis system (Olympus DP71). Quantitative histomorphometric analysis was conducted in a blinded fashion using Image-Pro Plus software, version 6.0 (Media Cybernetics Inc.).

### *In vitro* 3-D co-culturing of human OEs and human adult dental pulp cells with BMP7 and/or EREG

The Transwell cell culture inserts (Fisher Scientific, Pittsburgh, PA, USA) and Matrigel^TM^ (BD Biosciences, San Jose, CA, USA) were prepared as previously described. Five groups were then established. For group 1, which served as the control group, a complex of Matrigel^TM^/OEs was seeded onto the Transwell membrane, and another complex of Matrigel^TM^/OEs was seeded on top. For the four other groups, a complex of Matrigel^TM^/adult dental pulp cells was first seeded onto the Transwell membrane, and the complex of Matrigel^TM^/OEs was then seeded on top. The co-culture complexes were maintained in a 37 °C incubator for 30 min to allow the Matrigel^TM^ to set. Then, KGM-2 medium was added to both the upper and bottom chambers of the Group 1 Transwell membrane. Concurrently, for the four other groups, KGM-2 medium was added to the upper chamber of the Transwell apparatus and DMEM was added to the bottom chamber. Co-culture complexes were cultured for 1 week before 1 mM CaCl_2_ was added to the KGM-2 medium. Subsequently, 10 nM dexamethasone, 50 μg/ml ascorbic acid and 10 mM b-glycerophosphate were added to the DMEM^35^ with 50 μg/ml ascorbic acid and 10 mM b-glycerophosphate; 100 ng/ml recombinant human BMP7 (PeproTech, Rocky Hill, NJ, USA) was added to the DMEM of Group 3; 10 ng/ml recombinant human EREG (PeproTech, Rocky Hill, NJ, USA) was added to the DMEM of Group 4; and both 100 ng/ml BMP7 and 10 ng/ml EREG were added to the DMEM of Group 5. The complex was co-cultured for an additional 8 weeks before further processing. To exclude the direct effect of BMP7 and EREG on epithelial cells, OEs were cultured alone in the Transwell apparatus, and BMP and/or EREG were added into the culture. To elucidate the effect of BMP7 and/or EREG on odontogenic-associated gene expression in dental pulp cells, the dental mesenchymal cell culture was supplemented with BMP and/or EREG. Complexes from all groups were harvested, and RNA was collected using an RNeasy Mini kit (Qiagen, Valencia, CA, USA) following the manufacturer’s instructions. Then, cDNA template was synthesized using the SuperScript^TM^ III First-Strand Synthesis System (Invitrogen, Carlsbad, CA, USA), and qPCR was used to quantify the gene-expression levels of KRT14 (cytokeratin 14), *amelogenin*, *ameloblastin* and *amelotin*.

The Matrigel^TM^/cell complexes were embedded in OCT compound (Sakura Finetek, Torrance, CA, USA) and then cryosectioned at a 10-μm thickness and stored at −80 °C. Immunofluorescence was performed as previously described.

### Statistical analysis

All of the experiments were performed in triplicate, and the results are expressed as the means and standard deviations (SDs). Student’s t-test was performed to examine significant differences between the treated and untreated groups. SPSS version 11.5 software (Statistical Package for the Social Sciences, SPSS Inc., Chicago, IL, USA) was used for all statistical analyses. Differences between means were considered significantly different when *p < 0.05 and **p < 0.01.

## Results

### Dental papilla cells induces odontogenic determination of OEs

**Figure 1:**
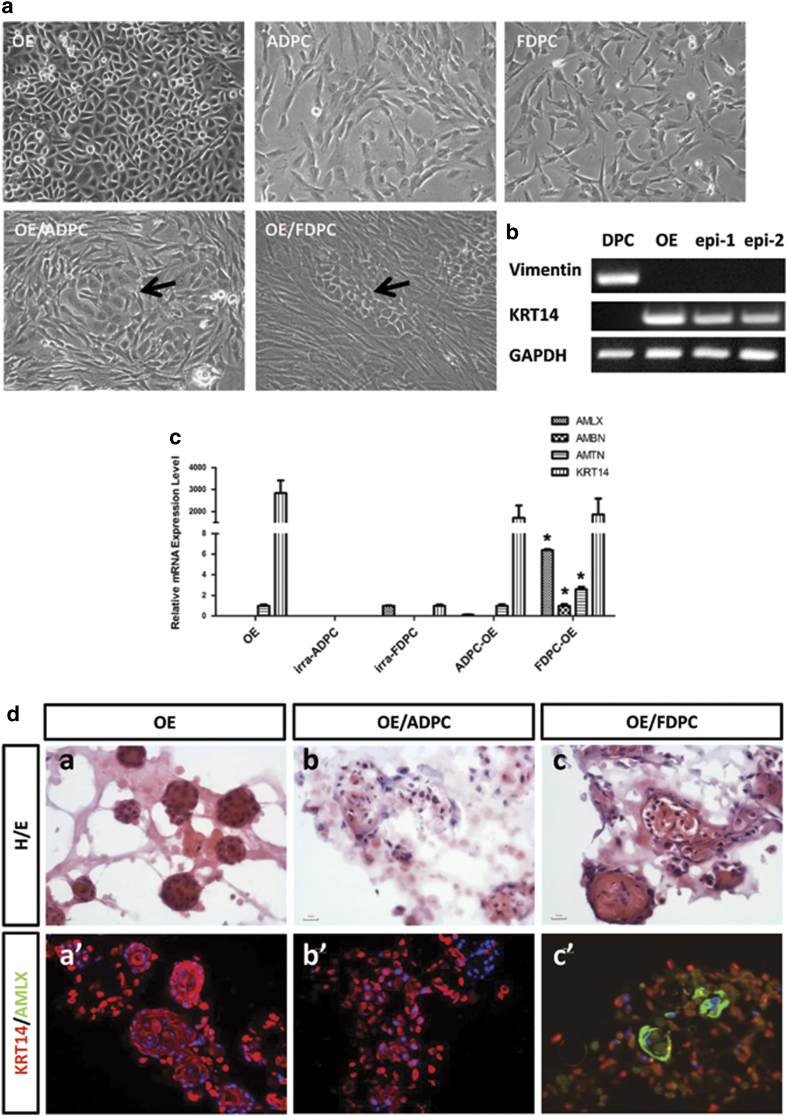
Oral epithelial cells (OEs) co-cultured with either fetal dental mesenchymal/papilla cells (FDPCs) or adult dental pulp cells (ADPCs). (**a**): Oral epithelial cells (OEs), irradiated adult dental pulp cells (ADPCs), and irradiated fetal dental papilla cells (FDPCs) were cultured *in vitro*. OEs were 2-dimensionally co-cultured with either ADPCs or FDPCs (OE/ADPC and OE/FDPC). OEs co-cultured with ADPCs and FDPCs formed colonies (arrow). (**b**): Co-cultured OEs (epi-1 and epi-2) expressed KRT14, while no vimentin expression was observed. (**c**): The relative mRNA expression levels of *amelogenin* (AMLX), *ameloblastin* (AMBN), *amelotin* (AMTN), and *cytokeratin 14* (KRT14) were detected by qPCR. OEs co-cultured with FDPCs had significantly higher expression levels of AMLX, AMBN, and AMTN than those co-cultured with ADPCs. Y-axis: fold change in gene expression. Scale bar: 50 μm. *, p < 0.01. (**d**): FDPCs promote the ameloblast-lineage differentiation of OEs under 3-D co-culturing conditions. OEs cultured alone under 3-D conditions in Matrigel^TM^ formed solid cell clusters (a) that stained extensively for KRT14 but that showed no positive signal for AMLX (a’). OEs co-cultured with ADPCs under 3-D conditions in Matrigel^TM^ failed to form epithelial nests (b). Cells were stained positive for KRT14 but not for AMLX (b’). OEs co-cultured with FDPCs under 3-D conditions in Matrigel^TM^ formed glandular structures (c). OE cells showed positive signals for both KRT14 and AMLX (c’). Scale bar: 20 μm.

OEs, ADPCs, and FDPCs were cultured *in vitro* (Fig. 1a, OE, ADPC, and FDPC). When co-cultured with mitosis-inactivated dental papilla cells, the OEs formed colonies on the mesenchymal feeder layer (Fig. 1a, OE/ADPC and OE/FDPC, indicated by arrows). The co-cultured OEs showed no vimentin expression, whereas K14 was expressed in these cells (Fig. 1b). The relative mRNA expression levels of *amelogenin*, *ameloblastin* and *amelotin* in the OEs co-cultured with dental papilla cells were markedly higher than in dental papilla cells cultured alone, and *KRT14* expression was down-regulated in the OEs when co-cultured (Fig. 1c). Under 3-D co-culturing conditions, the OEs formed a cell nest (Fig. 1da) that was solid and did not express amelogenin (Fig. 1da’). When co-cultured with adult dental pulp cells, the OEs did not show positive staining for amelogenin (Fig. 1db,b’), whereas when co-cultured with dental papilla cells, the OE nest formed an acinar structure (Fig. 1dc) and expressed amelogenin (Fig. 1dc’).

### Odontogenic gene expression differs in dental papilla cells and adult dental pulp cells

**Figure 2:**
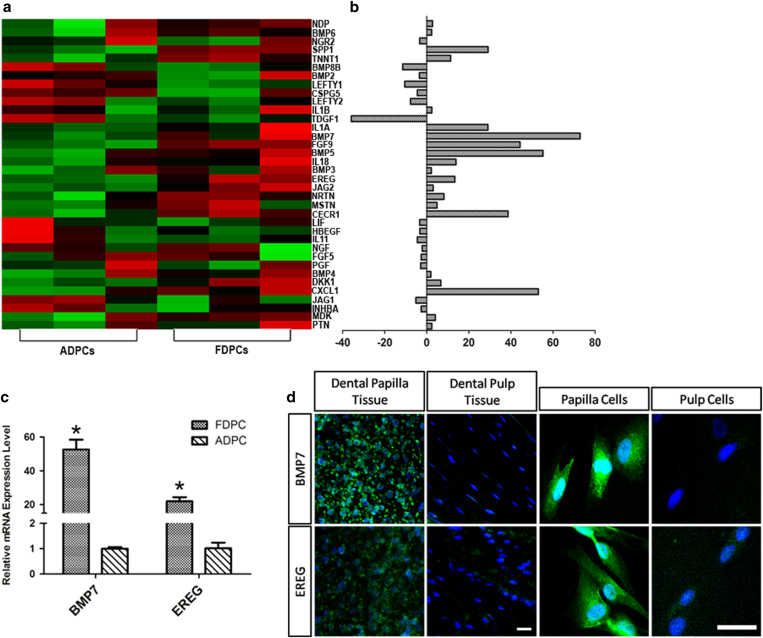
Growth factors were differentially expressed in FDPCs and ADPCs. (**a**) Heat map of differentially expressed (2.0-fold) growth factors between ADPCs and FDPCs (p < 0.05). (**b**) Bar graph showing the fold changes. (**c**) qPCR validation confirmed the differential expression levels of BMP7 and EREG between FDPCs and ADPCs. (**d**) BMP7 and EREG protein expression levels differed between dental papilla and adult dental pulp cells. *p < 0.01.

We used a human growth factor PCR array to ascertain whether growth factors that could be involved as odontogenic signals were differentially expressed in human dental papilla cells and human dental pulp cells. Heat maps and the fold differences in the differentially expressed growth factors in either human dental papilla cells or human dental pulp cells are presented in our results. We observed that dental papilla cells expressed significantly higher levels of BMP7 and EREG compared with adult dental pulp cells (p < 0.05) (Fig. 2a, b), and qPCR validation confirmed the significant differences in the expression levels of BMP7 and EREG between FDPCs and ADPCs (Fig. 2c). Remarkably more cells were positive for BMP7 and EREG in the dental papilla than in adult dental pulp (Fig. 2d).

### Amelogenin, ameloblastin and amelotin expression in OEs is induced by adult pulp cells treated with BMP7 and EREG

**Figure 3:**
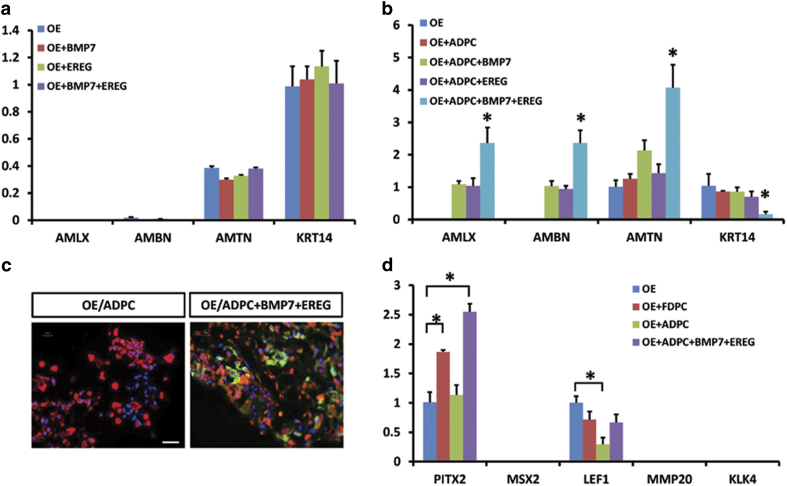
Supplementation of BMP7 and EREG to ADPCs induced the expression of AMLX, AMBN and AMTN in OEs. (**a**) BMP7 and EREG had no direct effect on the gene expression of oral epithelial cells. (**b**) qPCR detected the relative gene-expression levels under 3-D co-culturing conditions with different supplementation. OEs co-cultured with ADPCs without supplementation showed no AMLX expression. OEs co-cultured with ADPCs supplemented with BMP7 and EREG showed positive and significantly increased AMLX, AMBN, and AMTN expression. *KRT14* expression accordingly decreased after supplementation. (**c**) Immunofluorescence staining in OEs showed positive signals for amelogenin after BMP7 and EREG supplementation. Scale bar: 20 μm. (**d**) Odontogenic-associated transcriptional factors and mature markers of ameloblasts were tested. *PITX2* expression was significantly higher in OEs co-cultured with FDPCs and ADPCs supplemented with BMP7/EREG compared with OEs co-cultured with ADPCs. *LEF1* expression significantly decreased in OEs co-cultured with ADPCs.

To exclude the effect of BMP7 and EREG on oral epithelial cells, these two factors were supplemented into the lower chamber of the Transwell apparatus. After culturing, BMP7 and EREG had no effect on amelogenin, ameloblastin, amelotin, and KRT14 expression in OEs (Fig. 3a). The expression levels of amelogenin, ameloblastin and amelotin increased when OEs were 3-D co-cultured with dental pulp cells supplemented with BMP7 or EREG, while the KRT14 levels decreased (Fig. 3b). OEs co-cultured with adult dental pulp cells without supplementation stained positive for KRT14 but exhibited no amelogenin signal (Fig. 3c left panel). However, the co-culture supplemented with both BMP7 and EREG stained positive for both amelogenin and KRT14 (Fig. 3c right panel). BMP7 and EREG supplementation in ADPCs increased PITX2 expression in OEs compared with no supplementation (Fig. 3d). Decreased Lef1 expression in OEs co-cultured with ADPCs was rescued by supplementation with BMP7 and EREG (Fig. 3d).

### BMP7 and EREG promote the expression of PAX9 in adult dental pulp cells

**Figure 4:**
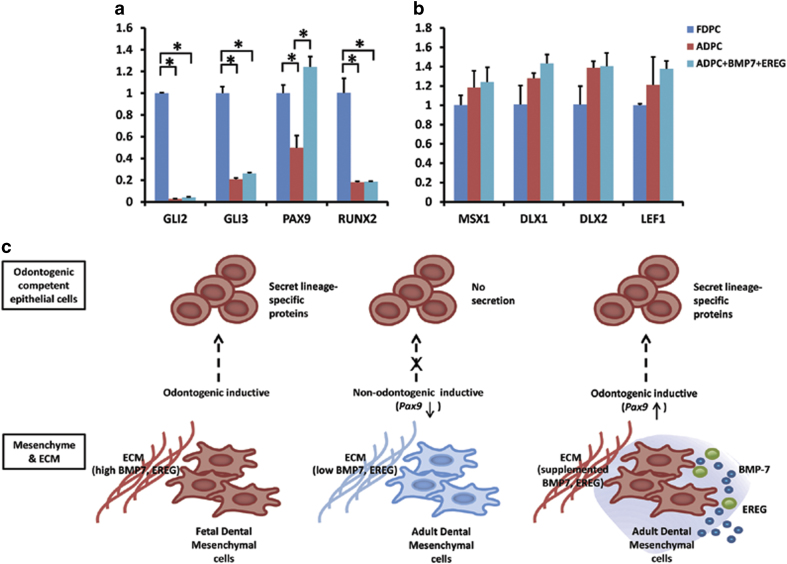
BMP7 and EREG contribute as niche factors to promote the inductive potential of dental mesenchyme. (**a**) *PAX9* expression decreased in adult dental pulp cells. Supplementation of BMP7 and EREG promoted *PAX9* expression in adult dental pulp cells. (**b**) Schematic of BMP7 and EREG contributions as niche factors to promote *PAX9* expression in dental mesenchyme, thus inducing the oral epithelial expression of odontogenic lineage-specific genes, such as amelogenin, ameloblastin, and amelotin.

To study the effects of BMP7 and EREG on adult dental mesenchyme, these two growth factors were supplemented into the culture of adult dental pulp cells. The relative mRNA expression levels of *Msx-1*, *Msx-2*, *Dlx-1*, *Dlx-2*, *Lef-1*, *Gli-2*, *Gli-3*, *Pax9*, and *Runx2* were examined. Although the levels of *Gli2*, *Gli3*, *Pax9*, and *Runx2* were significantly lower in ADPCs than in FDPCs (Fig. 4a), the levels of *Msx-1*, *Dlx-1*, *Dlx-2*, and *Lef1* were slightly, but not significantly, higher in ADPCs than in FDPCs (Fig. 4b). PAX9 levels were significantly upregulated in ADPCs after supplementation with BMP7 and EREG, and the mRNA expression levels were elevated to levels similar to those of FDPCs (Fig. 4a).

## Discussion

The present data address the critical barrier of a lack of viable postnatal cell sources for tooth regeneration in patients. The majority of tooth-regeneration studies have utilized either mouse dental cells at an early stage of morphogenesis or cells from human embryonic tooth germ, neither of which are viable cell sources for tooth regeneration in patients. In the present study, we compared the inductive potential and growth factor expression profiles of human fetal dental papilla cells and adult dental pulp cells. Human dental papilla cells at the late bell stage^31^,^36^ were used to induce non-dental oral epithelial cells. After induction, epithelial-specific *KRT14* expression was significantly down-regulated in OEs. This finding is consistent with previous studies on *KRT14* expression patterns in the developing tooth bud^37^,^38^ that have reported that *KRT14* expression decreases as ameloblasts differentiate. The finding that epithelial cells are capable of expressing *amelogenin*, *ameloblastin* and *amelotin* indicated the inductive potential of cultured dental papilla cells, even after thorough depletion of spatial information by monolayer culture and passage. The expression levels of amelogenin, ameloblastin and amelotin—three major extracellular enamel matrix proteins^39^,^40^,^41^—in non-dental oral epithelial cells after induction shed light on the possibility of inducing epithelial cells from other available sources into amelogenin-expressing cells. These findings increase our understanding of tooth regeneration.

Human dental pulp cells did not show similar inductive potentials and, unlike human dental papilla cells, did not induce enamel matrix protein gene expression in oral epithelial cells. Therefore, to explore the possible mechanisms underlying the differences in the inductive potential of ADPCs and FDPCs, we performed a comparative investigation of growth factor gene-expression levels. Of 36 growth factors that were differentially expressed between the two types of dental mesenchymal cells, EREG and BMP7 were chosen as candidate inductive signals of interest.

EREG, a member of the epidermal growth factor (EGF) family, stimulates cell growth, proliferation, and differentiation by binding to its receptor, EGFR^42^. More recently, the effect of EGF family members on tooth morphogenesis has been reported. Previous studies have shown that EGF superfamily molecules and their EGFRs are expressed during odontogenesis^43^. Several reports have indicated that EGF induces the proliferation of dental tissue and plays a key role in regulating initial inductive epithelial-mesenchymal interactions^44^,^45^,^46^,^47^. In addition, EGF down-regulation during early mouse development results in impaired tooth formation^48^. In our present study, FDPCs showed significantly higher EREG expression than ADPCs, suggesting that EREG may play a critical role in regulating inductive potential in the dental mesenchyme. Meanwhile, the observation that ADPCs supplemented with EREG and BMP7 induced the odontogenic commitment of non-dental epithelial cells suggests that EREG alone may not be able to regulate the inductive potential of the mesenchyme; rather, this regulation is dependent on other factors as well. Therefore, the odontogenic ability of these cells may rely on the effects of multiple growth factors, as defined in this study.

Five Bmp family molecules—*Bmp2*, *Bmp4*, *Bmp5*, *Bmp6* and *Bmp7*—were expressed at much higher levels in dental papilla cells than in dental pulp cells; the odontogenic capability of these proteins has been revealed in previous studies^49^,^50^,^51^,^52^,^53^. Some authors have noted that this family of proteins might prove useful in designing regenerative treatments for dental applications^53^,^54^. In particular, in the present study, a dramatic difference in the levels of *Bmp7* was observed between fetal and adult cells. Previous studies have indicated that *Bmp7* deletion affects the development of bones, teeth, and other ectodermal appendages of the orofacial complex^55^, and it has been confirmed that *Bmp7* can stimulate rat dentine-pulp complex formation *in vitro*^56^. One study also revealed that adenovirus-mediated BMP-7 expression can induce the odontogenic differentiation of human DPSCs and result in effectively mineralized tissue formation *in vivo*^50^. Our study indicated high expression levels of *Bmp7* in FDPCs, which may also suggest an essential role of *Bmp7* in cytodifferentiation and fate determination in tooth development. These findings may indicate that BMP7 is a central mediator of the epithelial-mesenchymal interactions that are necessary for correct tooth development and for the odontogenic ability of FDPCs.

We have previously shown that hESCs can be chemically induced to differentiate into ES-ECs that share similarities with dental epithelial cells^15^. Moreover, by co-culture with dental papilla cells *in vitro* and *in vivo*, ES-ECs can be further induced into ameloblast-lineage cells that express amelogenin^16^. In the future, adult dental pulp cells may be used in enamel regeneration following modification of their growth factor expression profiles (Fig. 4c) because these cells are more easily accessible than dental papilla cells. The present study indicated that *PAX9*, a gene belonging to the Pax family of homologous genes that code so-called paired-box-containing transcription factors, was expressed in fetal dental papilla cells and decreased in adult cells. The supplementation of BMP7 and EREG in adult dental pulp cells elevated *PAX9* expression and increased the inductive potential. This finding is in accordance with a previous study that showed that *Pax9* is expressed in neural-crest-derived mesenchymal cells^57^. *Pax9* null allele homozygotes resulted in arrested tooth formation after the bud stage, and the mesenchyme failed to condense around the growing epithelial bud. Evidence from that study also suggested that the knockout defect resides in the knockout mesenchyme, not in the epithelium^57^. It has been proposed that *Pax9* regulates the expression of Bmp4 in mesenchymal cells and is required for the expression of Msx1 and Lef1 after Bmp4 expression in the epithelium has ceased^57^. The elevated expression of *PAX9* in adult dental pulp cells after supplementation of BMP7 and EREG indicated increased inductive potential in these treated adult mesenchymal cells.

In addition to the essential functions of the multiple growth factor molecules, the extracellular matrix plays a very important role in cell-fate determination and differentiation^13^,^58^,^59^. The replication and osteogenic capabilities of aged mesenchymal stem cells can be rescued through exposure to young extracellular matrix^60^. In the present study, cells cultured in a monolayer were depleted of extracellular matrix by enzymes used during passage; however, these cells may secrete essential extracellular matrix proteins that contribute to the inductive potential. Whether exposure to fetal-like extracellular matrix can rescue the inductive ability of adult dental pulp cells or directly induce the odontogenic fate of non-dental epithelial cells will be of interest in our future research. This study and others have demonstrated conservation of the inductive potential in human dental tissues during early tooth development and will have implications for the next generation of stem-cell-based bioengineered human replacement teeth.

## Conclusions

In conclusion, *in vitro*-cultured late bell-stage fetal dental papilla cells retained and were able to induce non-dental oral epithelial cells to differentiate into an ameloblast lineage. The growth factor expression profiles were different in adult dental pulp cells, and these differences may have contributed to the deprivation of their inductive potential.

## Author Contributions

B.G. and X.Z. conceived and designed the experiments. X.Z. and L.W.Z. wrote the main manuscript text. B.G. and X.Z. prepared Figs. 1–4. CP and R.X. prepared Figs. 3 and 4. X.Z., M.W., J.Y., and Y.Z. performed the experiments. C.C.L. performed the bioinformatics analysis. J.X.S. and Y.Z. analyzed the data. X.D.Z. and L.W.Z. contributed reagents/materials/analytical tools. All authors reviewed the manuscript.

## Additional Information

**How to cite this article**: Gao, B. et al. BMP7 and EREG Contribute to the Inductive Potential of Dental Mesenchyme. *Sci. Rep.* **5**, 9903; doi: 10.1038/srep09903 (2015).
